# A cohort study investigating the occurrence of differences in care provided to men and women in an intensive care unit

**DOI:** 10.1038/s41598-021-02815-w

**Published:** 2021-12-03

**Authors:** Erik Zettersten, Gabriella Jäderling, Max Bell, Emma Larsson

**Affiliations:** 1 Perioperative Medicine and Intensive Care, Karolinska University Hospital, Solna, Stockholm, Sweden; 2 Section of Anaesthesiology and Intensive Care Medicine, Department of Physiology and Pharmacology, Karolinska Institute, Stockholm, Sweden

**Keywords:** Epidemiology, Outcomes research

## Abstract

It has been reported that there are differences in the care given within the intensive care unit (ICU) between men and women. The aim of this study is to investigate if any differences still exist between men and women regarding the level of intensive care provided, using prespecified intensive care items. This is a retrospective cohort study of 9017 ICU patients admitted to a university hospital between 2006 and 2016. Differences in use of mechanical ventilation, invasive monitoring, vasoactive treatment, inotropic treatment, echocardiography, renal replacement therapy and central venous catheters based on the sex of the patient were analysed using univariate and multivariable logistic regressions. Subgroup analyses were performed on patients diagnosed with sepsis, cardiac arrest and respiratory disease. Approximately one third of the patients were women. Overall, men received more mechanical ventilation, more dialysis and more vasoactive treatment. Among patients admitted with a respiratory disease, men were more likely to receive mechanical ventilation. Furthermore, men were more likely to receive levosimendan if admitted with cardiac arrest. We conclude that differences in the level of intensive care provided to men and women still exist.

## Introduction

Historically, it has been reported that more men than women are treated in the Intensive Care Unit (ICU)^1–5^ and that once admitted, men receive more mechanical ventilation, vasoactive drugs, renal replacement therapy (RRT) and invasive monitoring^2,3^. Furthermore, there are indications that men have a longer length of stay (LOS) in the ICU^5^. These reported differences do not seem to translate into a survival benefit for men, previous studies have shown conflicting results with no clear sex difference between men and women in survival^1,5,6^. It is not fully understood why gender discrepancies in ICU-treated patients exist. It is proposed that sex hormones have an impact on how the severity of an illness progresses, where female sex hormones are suggested to have a protective effect, showing lower risk of sepsis and better outcome after major bleeding and trauma in animal models^7–9^. That could possibly be a part of the explanation to the male dominance in ICU populations; women don’t develop critical illness in the same manner as men. However, data regarding the influence of sex hormones on the course of illness are conflicting^10,11^. We do not have evidence that medical care given in the ICU should be tailored after the sex of the patient. Then again, there is no clear evidence that the medical care should *not* be tailored after the sex of the patient. Therefore, differing care provided to men and women is still controversial. If differences exist, it has to be ruled out that it is caused by gender bias, unintentional or not. The question remains as to why men have an apparent greater need of intensive care, as indicated by the larger proportion of men in the ICU. Our group has previously investigated the afferent arm, i.e., if there are any differences in admittance to the ICU^12,13^, and the efferent arm, i.e., mortality and discharge patterns from the ICU^6^ in an effort to understand the ICU sex ratio. For this study, we aimed at investigating if there were any differences in the intensity of intensive care in a cohort of more than 9000 patients cared for at a university hospital. Our hypothesis is that given equal disease burden, men receive more intensive care in the ICU.

## Methods

### Setting and study population

This retrospective cohort study was conducted at a mixed 13-bed ICU at the Karolinska University Hospital, Stockholm, Sweden. Medical and surgical patients are treated in this ICU, including trauma patients. Cardiothoracic, neurosurgical and paediatric patients are treated in separate ICUs and not included in the current study. Eligible for inclusion were patients ≥ 18 years old treated in the ICU between January 2006 and December 2016. After exclusion of patients < 18 years (n = 234) and patients not found when cross-matching with Karolinska Database (KARDA) (n = 555 of which 36.6% were women) 9017 patients were included in the analysis. Our research group has previously analysed this dataset with the aim of investigating length of stay and 30- and 90-day mortality^6^.

### Data collection

All patients are registered in the electronic ICU patient data management system (PDMS, Clinisoft, GE Healthcare) from which we extracted data on admission diagnosis codes, Acute Physiology And Chronic Health Evaluation (APACHE II) score, Simplified Acute Physiology Score (SAPS 3), all codes for any therapy given within the ICU (invasive ventilation, invasive monitoring, vasoactive medication and renal replacement therapy) as well as time of admission, discharge and readmissions. Demographic data, comorbidities and 30- and 90-day mortality were extracted from KARDA.

### Outcomes

Primary outcome was sex difference in any of the items decided a priori as proxy for intensity of intensive care. The following items were included: invasive mechanical ventilation, invasive monitoring, central venous catheter placement, renal replacement therapy, echocardiography, presence of one vasoactive or inotropic drug and presence of more than one vasoactive or inotropic drug. Each outcome is considered separately.

### Definitions

Sex is broadly defined as either of the two main categories (male and female) into which humans and most other living species are divided on the basis of their reproductive functions. Gender is defined by the World Health Organisation as the socially constructed roles, behaviours, activities and attributes that a given society considers appropriate for men and women^14^. For this study, we had access to the patient’s social security number, in which it is registered if the patient is defined as a man or a woman. All analyses are performed using the social security number, i.e. the gender of the patient. We aim at using the term sex when discussing biological differences between patients, and gender when discussing reasons for why patients are treated differently depending on whether they are defined as a man or a woman in their social security number. It is important to note that a person might change their social security number depending on how they identify themselves. Patients were assumed to be mechanically ventilated if they presented any of the codes for intubation orally or nasally, ventilator treatment, inhalation anaesthesia or high frequency oscillation ventilation. Invasive monitoring was defined as the presence of a pulmonary artery catheter and it was included as an item if any codes for pulmonary artery catheterization were present. Central venous catheter (CVC) was included as an item if the code for placement of a CVC was present, it was not included as an item if only code for use of CVC was present. Renal replacement therapy was assumed when codes for continuous renal replacement therapy were present, or when code for central dialysis catheter placement was present. Echocardiography codes included both transthoracic and transoesophageal echocardiography. Vasoactive medication included only norepinephrine and epinephrine as there is no significant use of other vasoactive agents at the Karolinska Hospital ICU. Inotropic medication included levosimendan and milrinone only, for the same reason. In 2010, Karolinska Hospital Solna replaced APACHE II with SAPS 3. We therefore calculated Estimated mortality risk (EMR) from the APACHE II^15^ and SAPS 3^16^ scores using their specific formulas to be a proxy for severity of illness at admission.

### Statistical analysis

Continuous variables were compared using Wilcoxon rank-sum test and presented as median and interquartile range (IQR). Comparison of categorical variables was performed using Pearson’s Chi-squared test. The association between men and women and use of intensive care resources was analysed using univariate and multivariable logistic regression. Variables in the multivariable model were selected a priori and included age, estimated mortality risk and Charlson Comorbidity Index (CCI). Subgroup analyses were performed on patients diagnosed with sepsis, cardiac arrest and respiratory disease. Results are presented as odds-ratio (OR) and corresponding 95% confidence interval.

### Ethics

The Regional Ethical Review Board in Stockholm, Sweden approved the study (Registration Number 2014/756-31/1). The invasion of privacy in this anonymized retrospective register-based study is considered to be acceptable in relation to the potential benefits the project may bring to these patient categories. Patient consent was therefore waived according to the ethical approval from the Regional Ethical Review Board in Stockholm, Sweden. All research was conducted in accordance with national guidelines and regulations.

## Results

### Patient demographics

In total 9017 patients were included in the study. There were more men than women admitted to the ICU, with a male dominance of 63.5%. Demographic data are presented in Table 1. Median [IQR] age (61 [41–72] vs. 59 [41–70]; p = 0.001) and estimated mortality risk (11.1 [3.1–29.9] vs. 9.9 [2.9–29.1]; p = 0.03) were statistically higher in women. Median [IQR] CCI was slightly higher in men (2 [2–4] vs. 2 [2–4]; p = 0.03). There was no statistical difference between women and men regarding SAPS 3 (n = 6359) or APACHE II (n = 4251) scores. According to admission diagnosis, women were more likely to be admitted with a respiratory disease (22.6% of the women vs. 18.2% of the men; p < 0.001), neurological disease (2.2% vs. 1.4%; p = 0.01), intoxication (5.5% vs. 4.4%; p = 0.018), sepsis (12.7% vs. 10.2%; p = 0.004) or other (7.4% vs. 5.5%; p = 0.000) whilst men were more likely to be trauma patients (30.0% of the men vs. 15.9% of the women; p = 0.000). A sensitivity analysis conducted on patients not found in Karda revealed similar age and gender distribution (men 62.7%, median [IQR] age 60 [46–70], women 37.2 median [IQR] age 63 [47–73]) compared to included patients.

**Table 1:** Patient characteristics.

	Women	Men	p^a^
Sex (n = 9017)	3289 (36.5)	5728 (63.5)	
Age median (IQR) (n = 9017)	61 (41–72)	59 (41–70)	0.001
**Age (%)**			
≤ 40	796 (24.2)	1375 (24.0)	
41–60	811 (24.7)	1631 (28.5)	
61–80	1344 (40.9)	2330 (40.7)	
81–90	309 (9.4)	375 (6.6)	
91–110	29 (0.9)	17 (0.3)	
CCI, median (IQR)	2(0–4)	2 (0–4)	0.03
**CCI categories**			
0	1059 (32.1)	1955 (33.9)	
1–2	981 (29.7)	1654 (28.7)	
3–4	481 (14.6)	889 (15.4)	
> 5	780 (23.6)	1268 (22.0)	
SAPS 3, median (IQR) (n = 6359)	55 (42–68)	54 (41–68)	0.73
APACHE II, median (IQR) (n = 4251)	11 (6–18)	11 (6–17)	0.59
EMR, median (IQR) (n = 8898)	11.1 (3.1–29.9)	9.9 (2.9–29.1)	0.03
**ICU diagnosis, No. (%)**			
Cardiovascular	490 (14.8)	894 (15.5)	0.4^b^
Respiratory	746 (22.6)	1053 (18.2)	0.000^b^
Gastrointestinal	204 (6.2)	354 (6.1)	0.93^b^
Renal	53 (1.6)	100 (1.7)	0.65^b^
Sepsis/septic shock	419 (12.7)	617 (10.2)	0.004^b^
Neurological	71 (2.2)	82 (1.4)	0.01^b^
Trauma	526 (15.9)	1731 (30.0)	0.000^b^
Intoxication	183 (5.5)	256 (4.4)	0.018^b^
Other	246 (7.4)	316 (5.5)	0.000^b^

Continuous parameters presented as median (IQR). Categorical parameters presented as number (%) unless indicated otherwise.

*CCI Charlson Co-morbidity Index; SAPS 3* Simplified Acute Physiology Score; *APACHE II* Acute physiology and chronic health evaluation; *IQR* Interquartile Range; *ICU* Intensive care unit; *EMR* Estimated Mortality Risk.

^a^Wilcoxon rank-sum test, ^b^Chi-square test.

### Intensity of intensive care

Overall, men were more likely to receive mechanical ventilation [OR 1.28 (95% CI 1.17–1.41)], vasoactive treatment [OR 1.16 (95% CI 1.06–1.27)] and RRT (intermittent haemodialysis or continuous renal replacement therapy) [OR 1.21 (95% CI 1.04–1.40)] (Table 2). In additional subgroup analyses, men were more likely to be administered levosimendan if admitted to ICU with sepsis [OR 1.45 (95% CI 1.03–2.04)] or cardiac arrest [OR 2.11 (95% CI 1.27–3.49)]. If admitted with a cardiac arrest diagnosis, men more often received a central venous line [1.60 (95% CI 1.04–2.45)] (Tables 3 and 4). Men were also more likely to receive mechanical ventilation [1.22 (95% CI 1.01–1.49)] if admitted with a respiratory diagnosis (Table 5). Women were not more likely to receive any of the items investigated.

**Table 2:** Univariate and multivariable logistic regression analyses on all patients, exploring differences in the use of invasive mechanical ventilation, invasive monitoring, vasoactive treatment, echocardiography, RRT and placement of central catheters, based on the sex of the patient. Data presented as odds ratio and 95% CI.

	Univariate^a^	Multivariable^b^
**Invasive mechanical ventilation**		
Women	Ref	Ref
Men	1.27 (95% CI 1.16–1.38)	1.28 (95% CI 1.17–1.41)
**Invasive monitoring**		
Women	Ref	Ref
Men	1.09 (95% CI 0.81–1.48)	1.09 (95% CI 0.80–1.49)
**Vasoactive treatment**		
Women	Ref	Ref
Men	1.12 (95% CI 1.02–1.21)	1.16 (95% CI 1.06–1.27)
**Echocardiography**		
Women	Ref	Ref
Men	0.96 (95% CI 0.87–1.06)	0.96 (95% CI 0.87–1.06)
**RRT**		
Women	Ref	Ref
Men	1.22 (95% CI 1.06–1.41)	1.21 (95% CI 1.04–1.40)
**Central catheters**		
Women	Ref	Ref
Men	1.04 (95% CI 0.95–1.13)	1.09 (95% CI 0.99–1.19)

^a^9067 and 8863 patients are included in univariate analyses and multivariable analysis respectively.

^b^Adjusted for Age, Estimated mortality risk and Charlson Co-morbidity index.

*RRT* Renal replacement therapy.

**Table 3:** Univariate and multivariate logistic regression analyses on patients admitted with sepsis, exploring differences in the use of invasive mechanical ventilation, invasive monitoring, vasoactive treatment, echocardiography, RRT and placement of central catheters, based on the sex of the patient. Data presented as odds ratio and 95% CI.

	Univariate^a^	Multivariable^b^
**Invasive mechanical ventilation**		
Women	Ref	Ref
Men	1.01 (95% CI 0.79–1.30)	1.00 (95% CI 0.77–1.29)
**Invasive monitoring**		
Women	Ref	Ref
Men	1.10 (95% CI 0.67–1.83)	1.12 (95% CI 0.67–1.88)
**Vasoactive treatment**		
Women	Ref	Ref
Men	1.18 (95% CI 0.87–1.59)	1.12 (95% CI 0.82–1.53)
**Echocardiography**		
Women	Ref	Ref
Men	0.82 (95% CI 0.64–1.06)	0.77 (95% CI 0.59–1.00)
**RRT**		
Women	Ref	Ref
Men	1.27 (95% CI 0.96–1.68)	1.22 (95% CI 0.91–1.65)
**Central catheters**		
Women	Ref	Ref
Men	0.97 (95% CI 0.67–1.40)	0.87 (95% CI 0.59–1.28)
**Levosimendan**		
Women	Ref	Ref
Men	1.44 (95% CI 1.03–2.00)	1.45 (95% CI 1.03–2.04)

^a^1036 and 1007 patients are included in univariate analyses and multivariable analyses respectively.

^b^Adjusted for Age, Estimated mortality risk and Charlson Co-morbidity index.

*RRT* Renal replacement therapy.

**Table 4:** Univariate and multivariate logistic regression analyses on patients admitted with cardiac arrest, exploring differences in the use of invasive mechanical ventilation, invasive monitoring, vasoactive treatment, echocardiography, RRT and placement of central catheters, based on the sex of the patient. Data presented as odds ratio and 95% CI.

	Univariate^a^	Multivariable^b^
**Invasive mechanical ventilation**		
Women	Ref	Ref
Men	1.24 (95% CI 0.59–2.58)	1.16 (95% CI 0.54–2.50)
**Invasive monitoring**		
Women	Ref	Ref
Men	1.67 (95% CI 0.54–5.14)	1.72 (95% CI 0.55–5.33)
**Vasoactive treatment**		
Women	Ref	Ref
Men	1.46 (95% CI 0.94–2.26)	1.50 (95% CI 0.96–2.33)
**Echocardiography**		
Women	Ref	Ref
Men	1.14 (95% CI 0.79–1.65)	1.16 (95% CI 0.80–1.68)
**RRT**		
Women	Ref	Ref
Men	1.32 (95% CI 0.74–2.34)	1.39 (95% CI 0.77–2.50)
**Central catheters**		
Women	Ref	Ref
Men	1.59 (95% CI 1.04–2.42)	1.60 (95% CI 1.04–2.45)
**Levosimendan**		
Women	Ref	Ref
Men	2.04 (95% CI 1.24–3.37)	2.11 (95% CI 1.27–3.49)

^a^536 and 529 patients are included in univariate analyses and multivariable analyses respectively.

^b^Adjusted for Age, Estimated mortality risk and Charlson Co-morbidity index.

*RRT* Renal replacement therapy.

**Table 5:** Univariate and multivariate logistic regression analyses on patients admitted with respiratory disease, exploring differences in the use of invasive mechanical ventilation, invasive monitoring, vasoactive treatment, echocardiography, RRT and placement of central catheters, based on the sex of the patient. Data presented as odds ratio and 95% CI.

	Univariate^a^	Multivariable^b^
**Invasive mechanical ventilation**		
Women	Ref	Ref
Men	1.25 (95% CI 1.03–1.50)	1.22 (95% CI 1.01–1.49)
**Invasive monitoring**		
Women	Ref	Ref
Men	1.21 (95% CI 0.55–2.65)	1.11 (95% CI 0.49–2.47)
**Vasoactive treatment**		
Women	Ref	Ref
Men	1.19 (95% CI 0.99–1.44)	1.12 (95% CI 0.91–1.38)
**Echocardiography**		
Women	Ref	Ref
Men	0.98 (95% CI 0.80–1.21)	0.90 (95% CI 0.72–1.12)
**RRT**		
Women	Ref	Ref
Men	1.57 (95% CI 1.10–2.23)	1.39 (95% CI 0.97–2.01)
**Central catheters**		
Women	Ref	Ref
Men	1.06 (95% CI 0.88–1.28)	0.99 (95% CI 0.80–1.21)

^a^1799 and 1769 patients are included in univariate analyses and multivariable analyses respectively.

^b^Adjusted for Age, Estimated mortality risk and Charlson Co-morbidity index.

*RRT* renal replacement therapy.

## Discussion

The results from this cohort study of 9017 ICU patients indicate that men received more intensive care in the ICU, given equal burden of disease to women at admission. The causes for this are unclear, but men might have a more severe trajectory in their course of disease, as compared to women. In general, men received more mechanical ventilation, vasoactive treatment and RRT. When performing subgroup analyses we found that men admitted with a respiratory diagnosis received more mechanical ventilation, and that men admitted with sepsis or cardiac arrest received more inotropic treatment in the form of levosimendan. Interestingly, none of the items investigated were more likely to be provided to women.

Similar results have been previously reported^1–3,17^. Most noteworthy is the work by Valentin^2^ and Fowler^3^, two large multicenter studies. Both concluded that women received less intensive care when in the ICU. Our group has previously investigated if the sex of the patient had any impact on physicians’ willingness to admit patients to the ICU. We found no evidence supporting patient sex to be a factor in admitting patients^12,13^. In a follow-up study we showed that given equal disease burden as women, men had a lower probability of being discharged from the ICU^6^. The question remains whether women would have a survival benefit if treated exactly in the same manner as men. There are plausible explanations as to why men receive slightly more intensive care than women. This will be discussed below.

### Mechanical ventilation

Men seem to follow a different path than women regarding the progression of sepsis and respiratory illness. It appears as if men develop more severe sepsis^18^ and respiratory illness^19^ compared to women. By now there is considerable evidence supporting the role of estrogen as a mediator in the production of proinflammatory cytokines, namely IL-1, IL-6 and TNF-α^20^ and at least there are plausible explanations for differences in development of acute lung injury (ALI) and acute respiratory distress syndrome (ARDS) where different pathways have been described, resulting in less barrier dysfunction and inflammation in female rat lungs^19^. Similar results have been presented for the development of sepsis, where men had increased levels of TNF-α and decreased levels of IL-10 as compared to women^21^.

### Vasoactive treatment

In this study we found that men received more vasoactive treatment compared to women. The reason for this is most likely multifactorial. One interesting explanation could be different vascular response to illness, mediated in part by estrogen. The effect of estrogen on the cardiovascular system is still not clearly understood, but there are interesting animal models showing improved heart function^22^ and differences in vasoreactivity. Li et al. have investigated vasoreactivity in healthy humans and rats and found that premenopausal women had a stronger responsiveness compared to men and postmenopausal women. These results were strengthened by their rat model, where they found that male rats lost more of their vascular reactivity compared to female rats following haemorrhagic trauma. Infusion of exogen 17-β estradiol increased MAP and animal survival^23^.

### Renal replacement therapy

We found that men received more RRT than women. This is in line with previous research. In a very large study of approximately 195,000,000 patients from the United Kingdom^24^, men were 2.2 times more likely to develop acute kidney injury (AKI). Neugarten et al. used intervention and procedure codes for hemodialysis and hemofiltration as proxy for AKI, which introduces the possibility that they in fact investigated discrepancies in utilization of dialysis between men and women and not development of AKI. Supporting their argument are several studies that report either no difference in initiation of RRT given equal level of AKI between men and women, or that women were more likely to receive RRT^25–27^. Furthermore, there are compelling animal models supporting a protective role of estrogen in development of AKI after severe illness^28–30^.

### Inotropic treatment

Levosimendan is a positive inotropic drug primarily indicated for short term use in patients with heart failure and acutely diminished heart function. Its role in sepsis has been disputed recently^31,32^, but in selected cases it can still be a part of the treatment arsenal. Its use following cardiac arrest is still controversial, but animal studies suggest a beneficial effect^33^. So why then do men receive more levosimendan following cardiac arrest? One possible explanation could be that it is more common for men to develop macrovascular coronary artery disease, commonly leading to heart failure with reduced ejection fraction (HFrEF) while women are more prone to develop endothelial inflammation, leading to microvascular dysfunction, which in turn leads to heart failure with preserved ejection fraction (HFpEF)^34^. Furthermore, Iorga et al. introduced a rat model where they managed to preserve ejection fraction in heart failure with the use of estrogen^22^. Similar results have been presented by Mizushiba et al. They found that administration of estradiol post hemorrhagic shock in rats restored cardiac index to sham levels^9^.

Data on survival post cardiac arrest are conflicting. It appears that women have a higher immediate death rate, but possibly a better long term outcome^35–37^. As of now it is unclear to us why levosimendan should not be used to the same extent in women as in men.

By this point it is clear that the care provided to men and women differs slightly. Our intention with this investigation is not to conclude that one group receives *better* treatment than the other. Instead, we are interested in whether the difference in care provided is driven by different needs. Dichotomizing on sex is most likely an oversimplification and it is clear that data is lost in this process. However, this is the foundation on which we need to be able to individualize treatment and tailor treatment in the ICU. The question is which (to this point unknown) variables that would help us reach further.

By utilizing two different databases we get a high-resolution dataset with over 9000 patients, allowing for subgroup analyses. A limitation is the fact that we did not have access to detailed measures of organ failure on admission, which potentially could explain differences in the interventions studied. Nor did we have access to the progression of organ failure in the patients. Sequential Organ Fail Assessment score would have been an aid in interpreting the results. Further studies should include an even higher resolution dataset, including data on day-to-day progression of disease and organ failure. Residual confounders can never be ruled out in a cohort study. This is a single center study, which has implications on the external validity.

## Conclusion

In this large ICU population, we found that men received more mechanical ventilation, vasoactive treatment, dialysis and inotropy as compared to women even after adjusting for available confounders. However, we did not have access to organ failure progression in this cohort, which is a major limitation. The reasons for differences in intensity of care provided between men and women might be explained by different biological mechanisms or degrees of organ dysfunction between men and women presenting with critical illness, but gender bias must also be acknowledged as a possible mechanism.

## Data Availability

Upon reasonable request and according to ethical approval.

## Abbreviations

ICU: Intensive Care Unit
LOS: Length of stay
CCI: Charlson Comorbidity Index
SAPS3: Simplified Acute Physiology Score 3
APACHE II: Acute Physiology And Chronic Health Evaluation II
SOFA: Sequential Organ Failure Assessment
KARDA: Karolinska Database
CVC: Central venous catheter
ALI: Acute lung injury
ARDS: Acute respiratory distress syndrome
MAP: Mean arterial pressure
AKI: Acute kidney injury
RRT: Renal replacement therapy

## Code availability

Upon reasonable request and according to ethical approval.
